# Life cycle evolution in the trilobites *Balangia* and *Duyunaspis* from the Cambrian Series 2 (Stage 4) of South China

**DOI:** 10.7717/peerj.15068

**Published:** 2023-04-10

**Authors:** Zhengpeng Chen, Yuanlong Zhao, Xinglian Yang, Jorge Esteve, Xiong Liu, Shengguang Chen

**Affiliations:** 1 College of Resources and Environmental Engineering, Guizhou University, Guiyang, Guizhou, China; 2 Guizhou Research Center for Palaeontology, Guiyang, Guizhou, China; 3 Key Laboratory of Karst Georesources and Environment (Guizhou University), Ministry of Education, Guiyang, Guizhou, China; 4 Departamento de Geodinámica, Estratigrafía y Paleontología, Facultad de Ciencias Geológicas, Geológicas, Universidad Complutense de Madrid, Madrid, Spain

**Keywords:** Trilobite, Life cycle, Ontogeny, Evolution, Cambrian

## Abstract

The evolution process can be reconstructed by tracking the changes in the dynamic characters of life cycles. A number of related trilobites from the Cambrian of South China provide additional information for the study of trilobite evolutionary patterns, which has been hampered by previous incomplete fossil record though. Here, *Balangia* and *Duyunaspis* represent related Cambrian oryctocephalid trilobites from South China, are comprehensively discussed over the ontogeny, and the results show that, from *B. balangensis* via *D. duyunensis* to *D. jianheensis*, their exoskeletal morphology shows a directional evolution. Based on the direction of evolutionary changes in the development of *Balangia* and *Duyunaspis*, we speculate that *Duyunaspis* likely evolved from *Balangia* instead of *Balangia* evolved from *Duyunaspis*, as was previously assumed. This inference is also supported by the phylogenetic tree. This research provides not only a better understanding of the mechanisms of evolution in trilobites, but also new insights for the relationship between developmental evolutionary changes and phylogeny in trilobites.

## Introduction

The incompleteness of the fossil record is widely distributed in strata, so species with lower strata may be younger than those with higher strata in evolutionary relationships (Paterson, Edgecombe & Lee, 2019). This impedes the exploration of biological evolution, particularly the studies of heterochrony that require the determination of ancestor-descendant relationship. Heterochrony is widely defined as a potential pattern of evolution (McKinney, 1988; McKinney & McNamara, 1991) and thought to be a dominant factor in trilobite evolution (McNamara, 1986), which makes the heterochronic evolution in oryctocephalid trilobites earn much attention (McNamara, Yu & Zhou, 2003; McNamara, Yu & Zhou, 2006). However, the main problem of heterochrony in trilobites (*e.g.*, McNamara, 1986; McNamara, Yu & Zhou, 2003; McNamara, Yu & Zhou, 2006) is the determination of sexual maturity, which is hard to be addressed for the lack of reliable evidence indicating the onset of sexual maturity (Hughes, Minelli & Fusco, 2006). In consequence, it seems difficult to justify the perspective proposed by McNamara, Yu & Zhou (2006) that *Balangia* may have evolved from *Duyunaspis* by paedomorphosis.

Phylogenetic tree provides a possible solution for the determination of ancestries (Smith, 1994). The construction of paleontological phylogenetic tree is established based on the morphological character, so the study of morphology is a necessary prerequisite for phylogenetic tree that cannot be ignored. In particular, the development of trilobites may provide information on evolution (Park & Choi, 2011). Some morphological characters like glabellar furrows are progressively effaced during the course of trilobite evolution (Fortey & Owens, 1997; Webster, 2009). The effacement in trilobites progressively developed during ontogeny, rarely occurring in the early growth stage (*e.g.*, Robison & Pantoja-Alor, 1968; Webster, 2009), and some trilobites are effaced as well in phylogeny (*e.g.*, Fortey & Owens, 1997; Cotton, 2001; Webster, 2009; Zhu, Hughes & Peng, 2013; Mergl & Kozák, 2016). At present, the effaced morphological characters of trilobite are not fully understood, which is caused by multiple factors (Fortey & Owens, 1997; Webster, 2009). One of the values of the effaced trilobite is that the early remained morphological characters may aid in fixing the phylogenetic position of the effacement group (Webster, 2009).

The effaced trilobites provide phylogenetic information, and certain morphological characters of trilobites appeared repeatedly throughout their history (Fortey & Owens, 1997). Thus, the appeared morphologic characters in trilobites may provide corresponding information regarding phylogeny. Unfortunately, the appeared trilobites have not received appreciable attention yet. The greatest obstacle to the study of evolution, such as effacement and appearance, is access to available fossils. However, the massive trilobite fossils preserved in continuous deposited Cambrian shales in South China provide incomparable advantage to studying evolution.

Here we conducted morphological analysis on two species of oryctocephalid trilobites *Duyunaspis* (*Duyunaspis duyunensis* Zhang & Qian in Zhou et al., 1977 and *Duyunaspis jianheensis* Chen et al., 2018) and the only one species of oryctocephalid trilobites *Balangia* (*Balangia balangensis* Qian, 1961), which are all produced from Balang or Tsinghsutung formations, Cambrian Series 2 Stage 4, South China. The evolutionary relationship between *Balangia* and *Duyunaspis* was explored in terms of ontogeny and speciation. This study provides significant clues for further research on the evolutionary mechanism and evolutionary developmental biology of oryctocephalid trilobites.

## Materials & Methods

**Figure 1:**
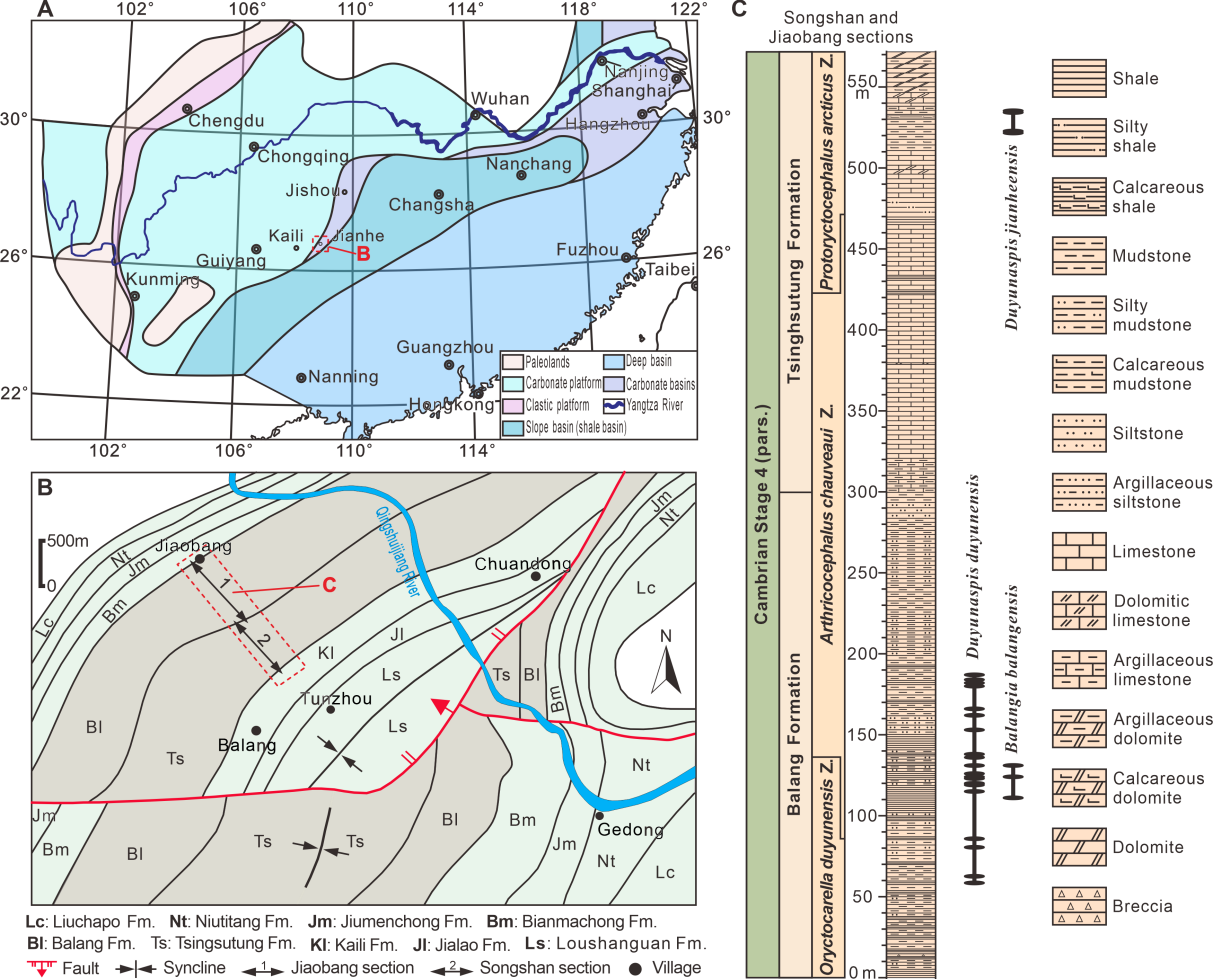
Location and geological setting of the Jiaobang and Songshan sections. (A) Lithofacies reconstruction of South China during the Cambrian Age 4. Based on Feng et al. (2001) and Zhang, Liu & Zhao (2008). (B) Geological map of the part of Jianhe County, Guizhou Province, China. The Jiaobang section and the Songshan section are two adjacent sections and can be linked together. (C) Stratigraphic occurrences of *Balangia balangensis*,* Duyunaspis duyunensis* and *D*. *jianheensis* in the Jiaobang and Songshan sections at Jiaobang and Balang villages , Jianhe County, Guizhou Province, China.

The oryctocephalid trilobites *Balangia* and *Duyunaspis* are currently found only on the Jiangnan Slope Belt of South China (Qian, 1961; Zhou et al., 1977; Zhang et al., 1980; Yuan, Zhao & Li, 2001; Yuan et al., 2002; McNamara, Yu & Zhou, 2006; Lei & Peng, 2014; Lei, 2016; Dai et al., 2017; Peng et al., 2017; Chen et al., 2018; Peng, 2020; Chen et al., 2022). *B*. *balangensis* and *D*. *duyunensis* occur in siliciclastic rocks of the Balang Formation (Fig. 1) in eastern Guizhou and western Hunan (Qian, 1961; McNamara, Yu & Zhou, 2006; Lei & Peng, 2014; Lei, 2016; Dai et al., 2017; Peng et al., 2017). *D*. *duyunensis* occurs in almost all of the Balang Formation in eastern Guizhou and western Hunan, by comparison, *B*. *balangensis* occurs only in a few localities (McNamara, Yu & Zhou, 2006; Peng, 2009; Wu et al., 2019), and it is difficult to find *B*. *balangensis* in the Balang Formation in western Hunan (Lei & Peng, 2014). *D*. *jianheensis* occurs in siliciclastic rocks from the Tsinghsutung Formation (Fig. 1) in eastern Guizhou (Chen et al., 2018; Chen et al., 2022). Both the Balang Formation (eastern Guizhou, southern Guizhou and western Hunan) and the Tsinghsutung Formation (eastern Guizhou) are thought to represent deep-water slope deposits (Peng, 2018). It is worth noting that, despite being at higher strata in fossil record, the number of *B*. *balangensis* is significantly smaller than that of *D*. *duyunensis*.

**Figure 2:**
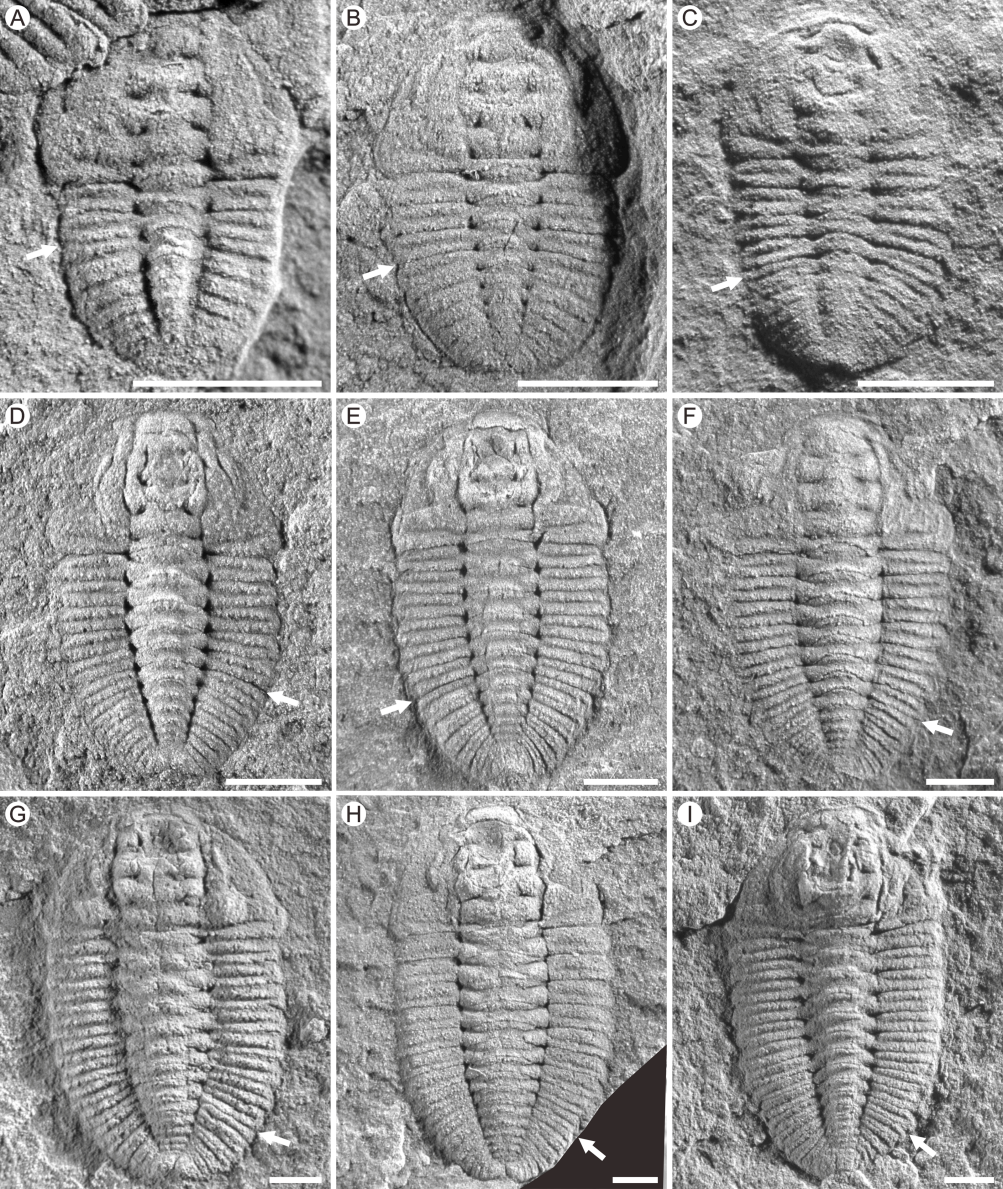
Photographs of *Duyunaspis jianheensis* from the Songshan section, Balang Village, Jianhe County, Guizhou Province, China. White arrows indicate the boundary between the thorax and the pygidium. (A) Meraspid degree two exoskeletons, Q51-2995. (B) Meraspid degree three exoskeleton , Q52-1462. (C) Meraspid degree four exoskeleton, Q52-2060B. (D) Meraspid degree five exoskeleton, Q51-1166. (E) Meraspid degree six exoskeletons, Q51-A2. (F) Meraspid degree seven exoskeleton, Q51-A3. (G) Meraspid degree eight exoskeleton, Q51-3202. (H) Meraspid degree nine exoskeleton, Q51-2823. (I) Holaspid exoskeleton, Q51-1834. All scale bars represent one mm. All the specimens are housed at the Guizhou Research Center for Palaeontology (GRCP).

**Figure 3:**
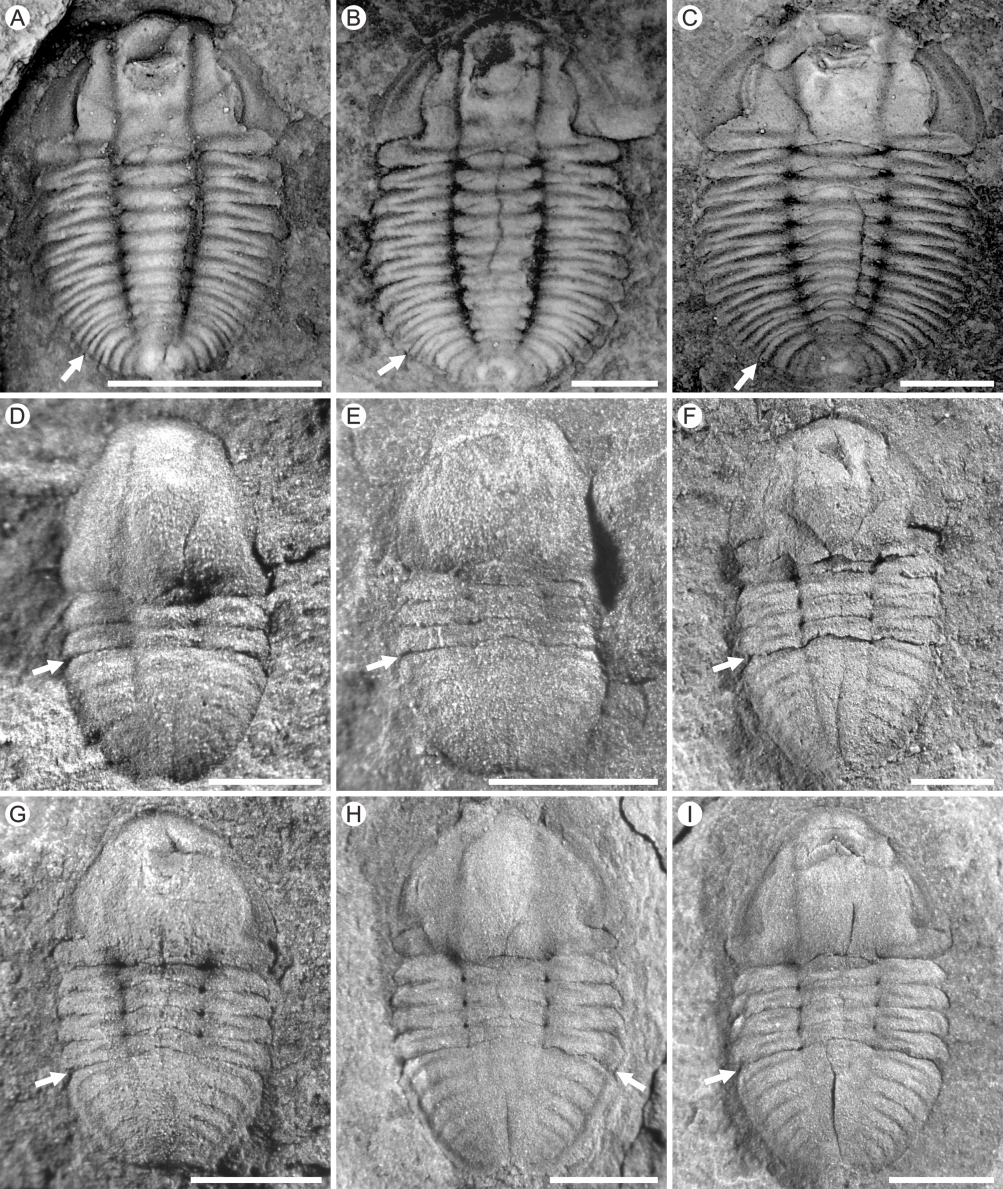
Photographs of *Duyunaspis duyunensis* and *Balangia balangensis*. (A–C) *Duyunaspis duyunensis* from the Zila A section (ZA) and Zila B (ZB), Zila Village, Huayuan County, Hunan Province, China (Lei & Peng, 2014; Lei, 2016). White arrows indicate the boundary between the thorax and the pygidium. (A) Meraspid degree eight exoskeleton, ZB, NIGP159520. (B) Meraspid degree eight exoskeleton, ZB, NIGP159755. (C) Meraspid degree nine exoskeleton, ZA, NIGP159527. (D–I) *Balangia balangensis* from the fossils site near the Jiaobang section, Jiaobang Village, Jianhe County, Guizhou Province, China. (D) Meraspid degree two exoskeleton, JJB-B-19-1. (E) Meraspid degree three exoskeleton, JJB-B-18-1; F, Holaspid exoskeleton, JJB-B-4-1. (G) Holaspid exoskeleton, JJB-B-13-1. (H) Holaspid exoskeleton, JJB-B-12-3. (I) Holaspid exoskeleton, JJB-B-12-1. All scale bars represent one mm except for D where the scale bars represent 0.5 mm. (A–C) courtesy of Qianping Lei. Specimens of *D*. *duyunensis* are housed at the Nanjing Institute of Geology and Palaeontology, Chinese Academy of Sciences (NIGP, CAS). Specimens of *B*. *balangensis* are housed at the Guizhou Research Center for Palaeontology (GRCP).

**Figure 4:**
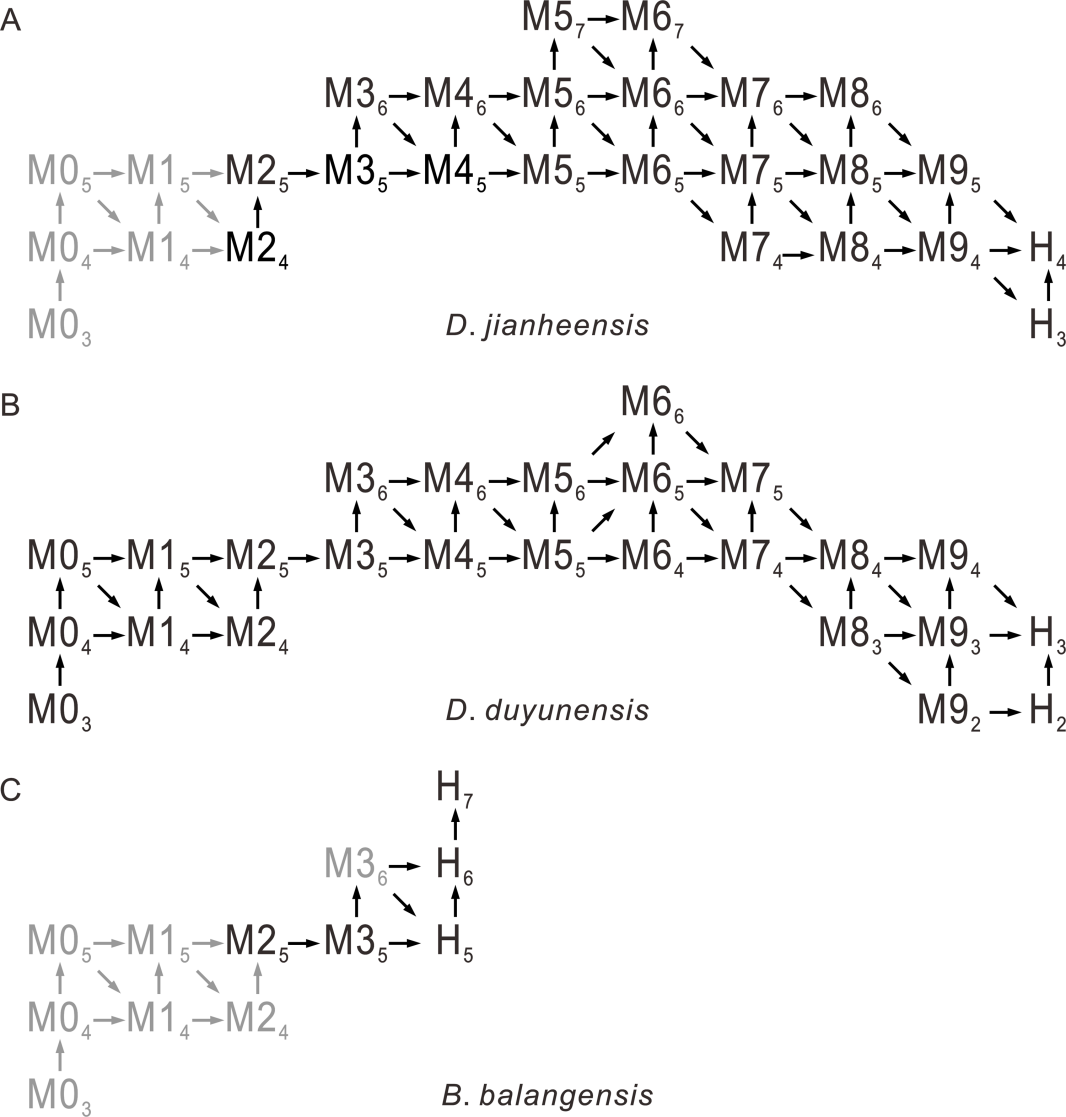
Possible ontogenetic of the segmentation pathways of *Duyunaspis jianheensis*, *D*. *duyunensis* and *Balangia balangensis*. Trilobite ontogenetic methodological standard from Hughes, Minelli & Fusco (2006) and Hughes et al. (2021) as reference. Gray indicates the speculative stage, in which no specimen has been collected for the time being. M designates meraspid instars and H designates holaspid instars. Mx_*y*_ (H_*y*_), where *x* is the number of thoracic segments and *y* the number of pygidial segments. The arrows indicate the direction of transition from different stages to subsequent stages. (A) *Duyunaspis jianheensis*, based on Chen et al. (2018) and the materials from this article. (B) *D*. *duyunensis* based on Dai et al. (2017). (C) *Balangia balangensis*, based on the materials from this article.

**Figure 5:**
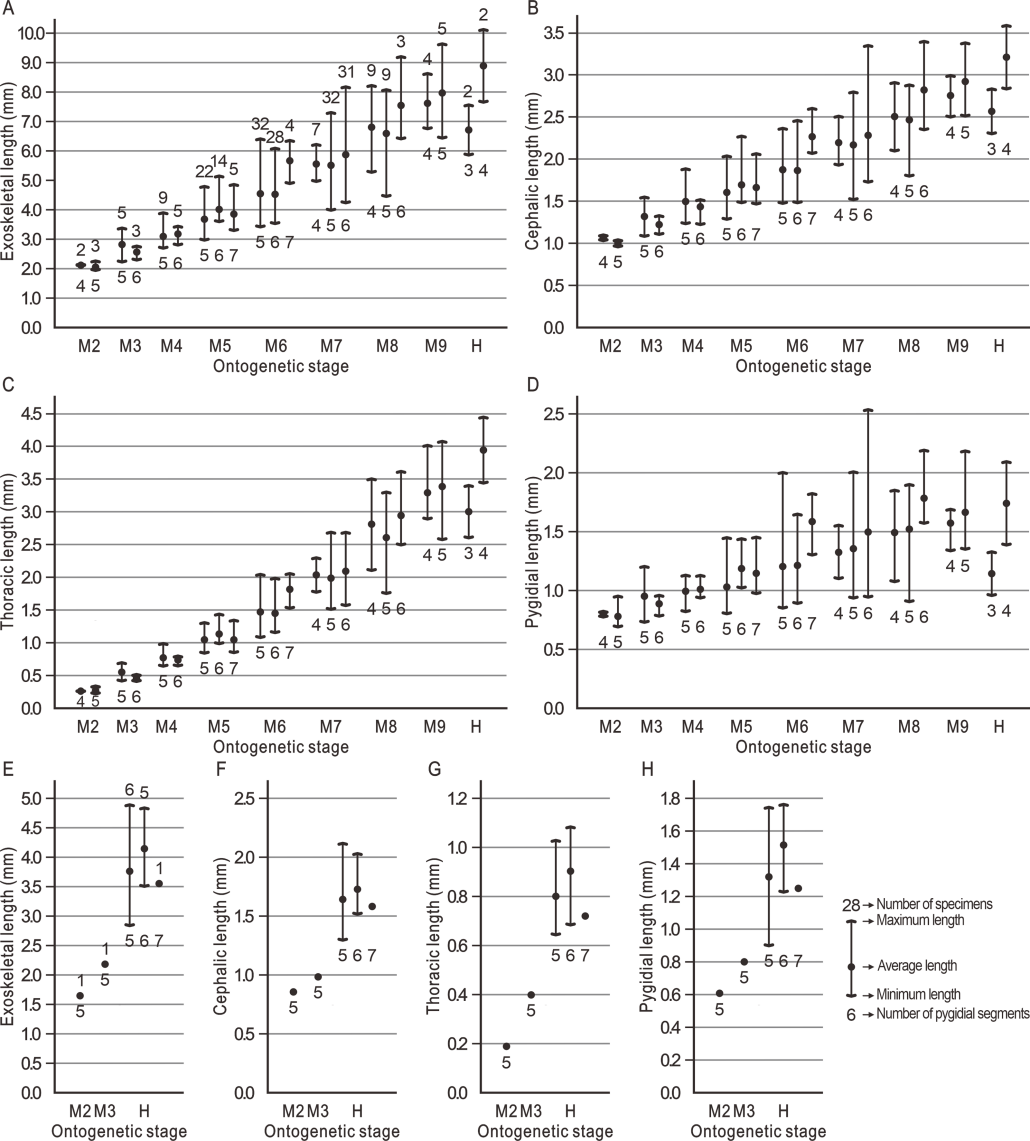
Body lengths of *Duyunaspis jianheensis* and *Balangia balangensis* in each period of ontogeny. (A–D) *Duyunaspis jianheensis* (see File S1). (E–H) *Balangia balangensis* (see File S2) .

A total of 236 specimens (see File S1) out of more than 1,000 specimens in *Duyunaspis jianheensis* (Fig. 2) were used in this study, and all were collected from the Songshan section in Balang Village, Jianhe County, Guizhou Province. A total of 14 specimens (see File S2) out of 21 specimens in *Balangia balangensis* (Figs. 3D–3I) were used, which collected from the fossils site near the Jiaobang section in Jiaobang Village, Jianhe County, Guizhou Province. *D*. *duyunensis* refers to Figures 2–5, 7 in Dai et al. (2017). All the specimens (except for the *D*. *duyunensis*) in this study are housed at the Guizhou Research Center for Palaeontology (GRCP). The phylogenetic position of *D*. *paiwuensis* is not discussed in this article due to its small number of specimens, unclear stratigraphic range (Lei & Peng, 2014) and its significantly different morphology from other species.

The specimens were photographed using a Leica DVM6 microscope. The resulting images were processed using the Digimizer software. Cephalic length means the length along the sagittal line from the anterior cephalic margin to the posterior occipital margin. Thoracic length means the length along the sagittal line from the occipital furrow to the anterior pygidial margin. Pygidial length means the length along the sagittal line from the anterior pygidial margin to the posterior pygidial margin. Exoskeletal length is the sum of the length of the cephalon, thorax and pygidium. All data were averaged after three measurements.

The morphological terms used herein follow Whittington (1997a). The ontogenetic pattern of trilobites is established based on suggestions by Hughes, Minelli & Fusco (2006) and Hughes et al. (2021). In addition, we use the term “appeared” to refer to morphological character that emerge gradually during the ontogeny of trilobite, as opposed to “effaced” (p. 265, Fortey & Owens, 1997).

## Results

In the ontogeny of *Balangia balangensis*, *Duyunaspis duyunensis* and *D*. *jianheensis*, the number of pygidial segments was not fixed during the same degree (with the same number of thoracic segments). The number of pygidial segments is present in two or three types at the same degree (Fig. 4). There were at most ten thoracic segments, six pygidial segments and fifteen trunk segments in the ontogenetic sequences of *D*. *jianheensis* (Fig. 4A)*.* There were at most ten thoracic segments, six pygidial segments and thirteen trunk segments in the ontogenetic sequences of *D*. *duyunensis* (Fig. 4B)*.* There were at most four thoracic segments, seven pygidial segments and eleven trunk segments in the ontogenetic sequences of *B*. *balangensis* (Fig. 4C)*.* Several instars, when only thoracic segments other than trunk segments increase, may exist in *B*. *balangensis*, *D*. *duyunensis* and *D*. *jianheensis* (Fig. 4). Although the number of the pygidial segments of *D*. *duyunensis* and *D*. *jianheensis* varied during the meraspid period, but the overall trend was decreasing, while the number of the pygidial segments of *B*. *balangensis* increased, observed in samples collected, all the postembryonic stages, including the holaspid period (Fig. 4).

During the ontogeny, the exoskeletal length of *Duyunaspis jianheensis* gradually increases and is longer than that of *D*. *duyunensis* (Dai et al., 2017) at the same period. In particular, during the M6–M8, the exoskeletal length of the morph with the most pygidial segments in the same meraspid degree is closer to that of the next meraspid degree (Fig. 5A). The cephalon of *D*. *jianheensis* increases gradually in length during ontogeny. During the M6–M8, the cephalon length of the morph with the most pygidial segments in the same meraspid degree is closer to that of the next meraspid degree (Fig. 5B). The thorax of *D*. *jianheensis* increases gradually in length during ontogeny. In the same meraspid period, the length of the thorax is almost identical at different morphs (Fig. 5C). The pygidium of *D*. *jianheensis* gradually increases in length during ontogeny. During the M6–M8, the pygidial length of the morph with the most pygidial segments in the same meraspid degree is closer to the pygidial length of the next meraspid degree (Fig. 5D). The length (including the exoskeleton, cephalon, thorax and pygidium) of *B*. *balangensis* all gradually increases during ontogeny (Figs. 5E–5H).

**Figure 6:**
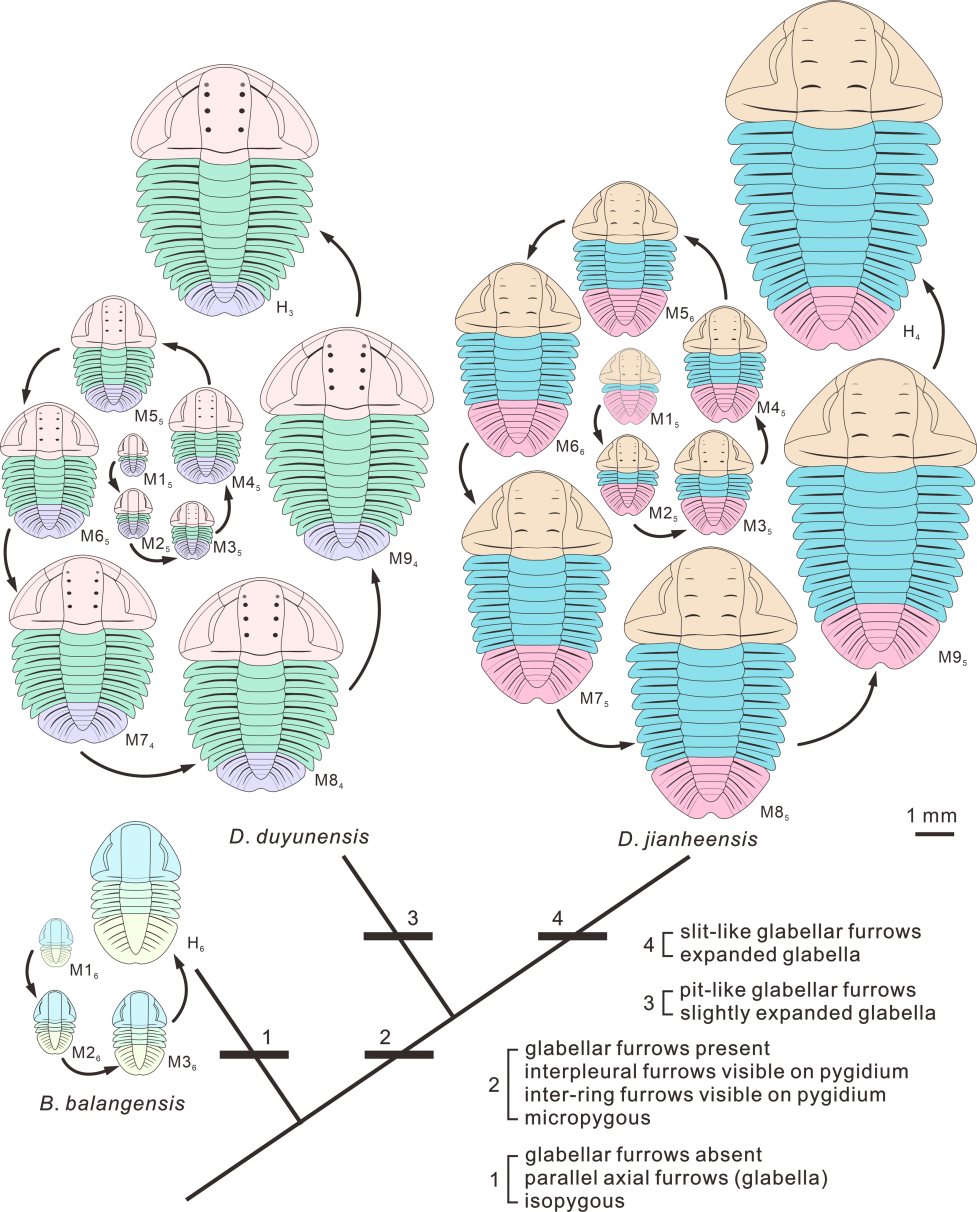
Evolutionary changes in development of the exoskeleton of *Balangia balangensis*, *Duyunaspis duyunensis* and *D*. * jianheensis*. The idealized ontogenetic pathway for each of the three species is one of those illustrated in Fig. 4. The number of pygidial segments in *Duyunaspis jianheensis* during the ontogeny is referenced to the mean number of pygidial segments (see Fig. 7). The number of pygidial segments in *D*. *duyunensis* and *Balangia balangensis* is referenced to *D*. *jianheensis*. Reconstructions in dorsal view of ontogeny of *D*. *jianheensis*, based on Chen et al. (2018) and the materials from this article, M1_5_ is speculative and no due to lack of specimens of it have been found. Reconstructions in dorsal view of ontogeny of* D*. *duyunensis*, based on Dai et al. (2017), the meraspid M9_4_ is speculative because the specimens were incomplete. Reconstructions in dorsal view of ontogeny of *B*. *balangensis*, based on the materials from this article (Figs. 2D–2I), M1_6_ is speculative and because of no specimens of it have been found. Grey glabellar furrows indicate indistinguishable or missing S4.

**Figure 7:**
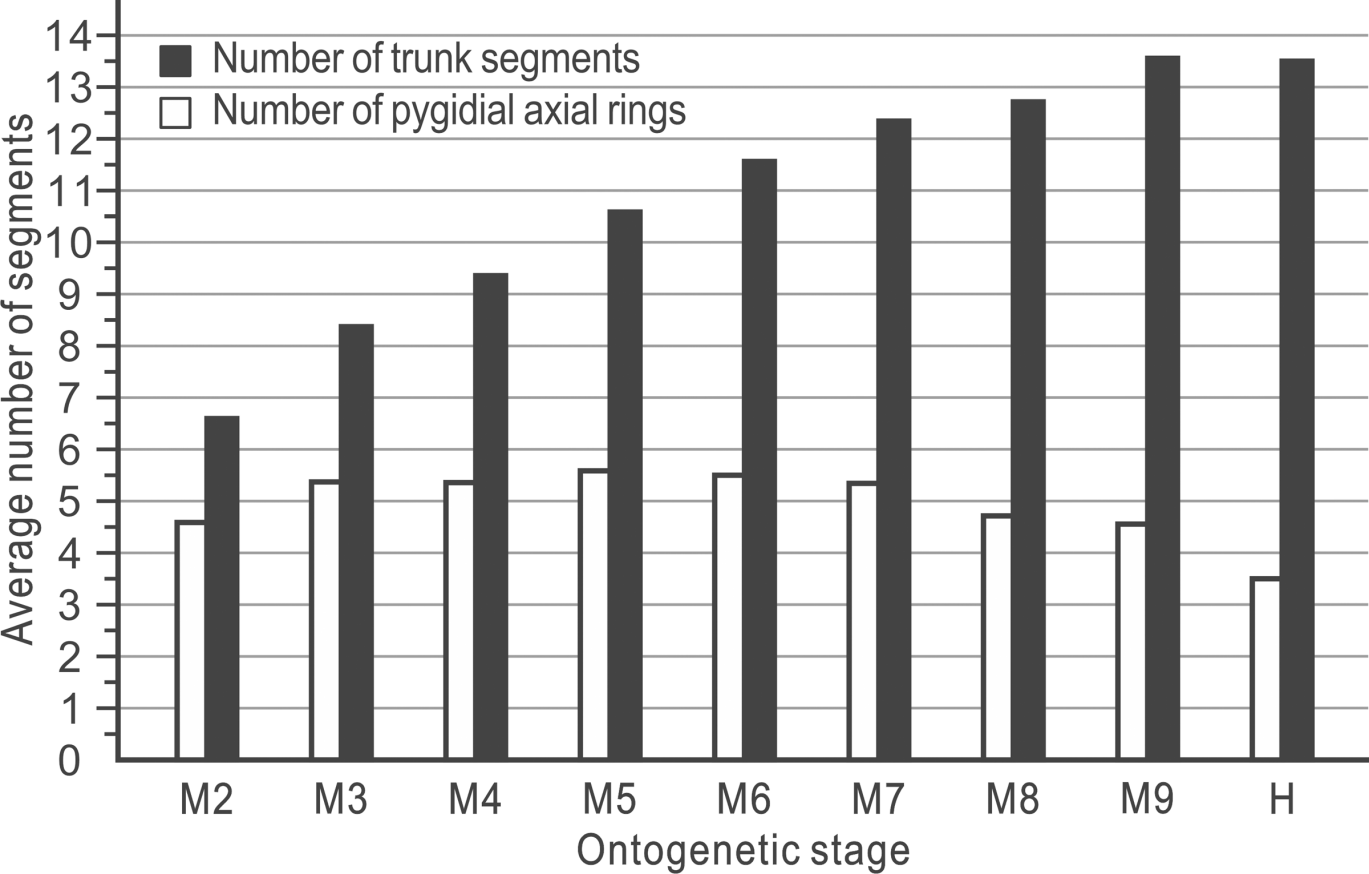
The mean numbers of trunk and pygidial segments for each ontogenetic stage of the *Duyunaspis jianheensis* (see File S1).

The ontogenetic pathways of *Balangia balangensis*, *Duyunaspis duyunensis* or *D*. *jianheensis* are probably multiple pathways (Fig. 4). Referring to Dai et al. (2021a); Dai et al. (2021b), an average ontogenetic pathway (Fig. 6) was derived by calculating the average pygidial segments of *D*. *jianheensis* (Fig. 7). Two segments were added in M4_5_ to M5_6_ in this pathway. This pathway is only one of many possibilities and may not represent the true ontogenetic pathway. The morphological characters of the morph with the most pygidial segments (except for exoskeletal, cephalic and pygidial length) are almost identical to those of the other morph in the same meraspid degree. Thus, the average ontogeny pathways for *D*. *jianheensis* can reflect the changes in morphological characters during ontogeny. The case of *B*. *balangensis* and *D*. *duyunensis* are similar to that of *D*. *jianheensis*. Based on that, possible ontogenetic pathways of *B*. *balangensis* and *D*. *duyunensis* are shown in Fig. 6. Similarly, the two pathways are only two of many possible pathways and may not represent the true ontogenetic pathways, but they can reflect the changes in morphological characters during ontogeny.

The size of pygidium was nearly equal to that of the cephalon during the early ontogeny (M2, Fig. 2A) for *Duyunaspis jianheensis*, which can approximately be regarded as isopygous. It could be inferred that it might be isopygous in the M1 (Fig. 6). Compared to the cephalon, the size of pygidium progressively narrowed down and finally became micropygous (holaspid) during the late ontogeny (Figs. 2B–2I and 6). The middle of the glabella gradually changed from slight to significant expansion during ontogeny. Despite the fourth pair of glabellar furrows (S4) being indistinguishable throughout the ontogeny, the glabellar furrow (S1–S3) and inter-ring furrow (pygidium) were developed and obvious. Since no specimens of the whole ontogenetic sequence have been obtained, it can only be confirmed that the glabellar furrow of *D*. *jianheensis* had already appeared in M2 and was pit-like (Figs. 2A, 6). The shape of glabellar furrows in *D*. *jianheensis* changed progressively from pit-like to slit-like during ontogeny. The facial suture type remained the same, belonging to proparian all the period (M2–H, Figs. 2 and 6). The anterior border was shorter (sag.) and preserved as linear shape throughout the ontogeny (Figs. 2 and 6).

In the early ontogeny of *Duyunaspis duyunensis* (Fig. 6), the size of pygidium was closely equal to that of the cephalon, which can be approximately regarded as isopygous. While in the late ontogeny of *D*. *duyunensis*, the size of the pygidium narrowed down progressively compared to the cephalon and finally turned to be micropygous in the holaspid period. The glabella gradually changed from parallel on both sides to slightly expand in the middle during the ontogeny. The glabellar furrows of *D*. *duyunensis* are barely developed or weak in the early ontogeny and only gradually emerged and deepened until the late degrees. At the holaspid period (H), *D*. *duyunensis* had a pit-like glabellar furrows. The fourth pair of glabellar furrows (S4) is indistinguishable throughout the ontogeny. Though unconspicuous in the early stage, the inter-ring furrow in the pygidium developed at the M1. The type of facial suture changes from proparian (M0–M6) to gonatoparian (M7–M8) and finally to opisthoparian (M9–H) (Fig. 6). The anterior border progressively became shorter (sag.) during the ontogeny and approximately linear in holaspid period (Fig. 6).

In the ontogeny, the pygidium of *Balangia balangensis* was nearly the same size as the cephalon, being isopygous (Figs. 3D–3I, 6). The glabella remains parallel on both sides during the ontogeny. The glabellar furrow, eye ridge and inter-ring furrow (pygidium) were barely developed. The type of facial suture in the early ontogeny (M2–M3, Figs. 3D–3F, 4C) was proparian, the posterior branch of the facial suture approached genal angle in late ontogeny (H, Figs. 3G–3I, 6), approximately gonatoparian suture. Anterior border was relatively narrow during the ontogeney.

## Discussion

Evolution is considered to be the change of ontogenies or life cycles over time instead of a simple modification of genotypes and phenotypes (Gilbert, Opitz & Raff, 1996; Fusco, 2019). As an important part of the life cycle, ontogenetic processes play a key role in evolutionary and developmental biology (*e.g.*, Jaeger, 2019). The reconstruction and comparison of ontogenetic series of extinct species provide important insights into the phylogenetic relationships of organisms and evolutionary changes in development (*e.g.*, Waloszek & Maas, 2005; Minelli & Fusco, 2008; Park & Choi, 2011; Fusco et al., 2012; Fusco, Hong & Hughes, 2016; Hughes et al., 2017; Holmes, Paterson & García-Bellido, 2021a. Because of the basal phylogenetic position and geological age of the underlying trilobites, their ontogeny is thought to potentially reveal ancestral characters of arthropod development (Hughes, Minelli & Fusco, 2006). The study of the selection and evolution of ontogenetic pathways in trilobites remains challenging (*e.g.*, Hughes, Minelli & Fusco, 2006; Hughes et al., 2021; Dai et al., 2021a; Dai et al., 2021b). The timing of the release of trilobite segment to the thorax and to segment expressed in the subterminal growth zone is sometimes not perfectly synchronized and varies by species (Hughes, Minelli & Fusco, 2006; Shen et al., 2014; Lei, 2016; Dai et al., 2017; Dai et al., 2021a; Dai et al., 2021b; Chen et al., 2018; Du et al., 2020; Holmes, Paterson & García-Bellido, 2021a). In some trilobites, especially the oryctocephalid trilobites (Lei, 2016; Dai et al., 2017; Dai et al., 2021a; Dai et al., 2021b; Chen et al., 2018; Du et al., 2020), there are degrees with the same number of thoracic segments but different numbers of pygidial segments. This casts doubt on the reconstruction of their ontogenetic pathways, as there are segments that are reabsorbed in a number of successive stages (Hughes et al., 2021; Dai et al., 2021b). Although a trilobite ontogenetic pathway based on segmental patterns has been proposed as a standard for trilobite ontogenetic pathway (Hughes et al., 2021), there are still many observations that have not been rationally explained yet. With more than 1,700 specimens, Dai et al. (2021b) made a discussion on the ontogenetic pathway of *Oryctocarella duyunensis* using statistical methods, and adopted an average state (average number of pygidial and trunk segments) to reconciled meraspid periods with different numbers of pygidial segments (Dai et al., 2021b). As Dai et al. (2021b) stated, “Our recognition of degrees and morphs is a descriptive one, and does not lead directly to any particular interpretation of ontogenetic stages and sequence.”

The postembryonic developmental stages of trilobites are delineated based on the development of articulations between segments (Hughes, Minelli & Fusco, 2006). A series of successive postembryonic developmental stages can be divided into protaspid, meraspid or holaspid periods based on the number of thoracic segments (Chatterton & Speyer, 1997; Fusco et al., 2012). The variation in the number of postembryonic stages within a species due to factors such as population density, food, light and temperature in living arthropods is known as developmental polymorphism (Beck, 1971; Solomon, 1973; Chippendale & Yin, 1973; Kfir, 1991; Quennedey et al., 1995; Esperk, Tammaru & Nylin, 2007; Minelli & Fusco, 2013). The diversity in the number of pygidial segments of oryctocephalid trilobites at the same degree may be the result of developmental polymorphism. One (only two different quantities of pygidial segments in this degree) or two (three different quantities of pygidial segments in this degree) morphs of the same degree of *Duyunaspis jianheensis* account for the majority in the sample (Fig. 5A). The oryctocephalid trilobite *Oryctocarella duyunensis* has same phenomenon (Dai et al., 2021b). So, the multiple ontogenetic pathways of oryctocephalid trilobites are obviously differ from the majority of trilobites that have only a single ontogenetic pathway (see Hughes, Minelli & Fusco, 2006). Degrees with the same thoracic segments but different pygidial segments are also observed in ptychopariid (Shen et al., 2014) and ellipsocephaloid (Holmes, Paterson & García-Bellido, 2021a) trilobites. To some degree, this indicates that the diversity in the number of pygidial segments for the same degree may not be an exceptional case in early trilobites. Any possible ontogenetic pathway selected (the sequence in the pathway is based on the number of thoracic segments) brings little effect on the description for morphological changes in *Balangia balangensis*, *Duyunaspis duyunensis* and *D*. *jianheensis* (see below), for these morphologic changes are progressive (with the thoracic segment increasing) rather than sudden. Here by using the ontogenetic pathway of Fig. 6, we make a discussion on evolutionary changes in development of the glabellar furrows, glabellar, anterior border, facial suture and relative size of pygidium and cephalon. This discussion also applies in the context of other possible ontogenetic pathways.

Some characters have appeared repeatedly over the course of trilobite evolution, such as the effacement (Fortey & Owens, 1997). The effaced characters (*e.g.*, glabellar furrow, occipital furrow, and axial furrow) are usually augmented during the ontogeny or clade of a group (Fortey & Owens, 1997; Webster, 2009). Webster (2009) suggested comparative morphology at early (pre-effacement) ontogenetic stages may therefore be useful in resolving phylogenetic placement of effaced taxa within otherwise non-effaced clades. Interestingly, the glabellar furrow in *Balangia balangensis*–*Duyunaspis duyunensis*–*Duyunaspis jianheensis* appeared and deepened progressively (Fig. 6). The presence or absence of the glabellar furrow is one of the main differences between *Balangia* and *Duyunaspis* according geometric morphometrics (Chen et al., 2022). The glabellar furrows of *B*. *balangensis* is absent and underdeveloped during the ontogeny (Figs. 3D–3I, 6). The glabellar furrows of *D*. *duyunensis* progresses from absent to present during the ontogeny and eventually becomes pit-like (Dai et al., 2017). The glabellar furrows of *D*. *duyunensis* collected from different lithologies and localities all exhibit the same case (McNamara, Yu & Zhou, 2006; Lei, 2016; Dai et al., 2017), ruling out the possibility that the glabellar furrows come from absence to presence due to compaction. The glabellar furrows of *D*. *jianheensis* gradually changes from pit-like to slit-like during the ontogeny (Figs. 2 and 6). This ontogenetic trend towards glabellar furrows is phylogenetically mirrored in a large part.

Though morphological function of the effaced character is unclear (Webster, 2009), they are not thought to be related to the living environment or the dorsal shell morphology (Fortey & Owens, 1997). Fortey & Owens (1997) suggested that multiple selection pressure can lead to effacement, which was beneficial to trilobites in holaspid period. To some extent, we speculate that the appearance may also be beneficial to trilobites in holaspid period. However, to date we are not sure if there is connection between the appearance of the glabellar furrow, the plasticity of the posterior branch of the facial suture (facial suture type) and the environment, the development of corresponding organs, or the changes in living habits. It is more likely that glabellar furrow did not develop in the ancestral taxa of *Balangia balangensis*. Conversely, it also possible that the ancestral taxon of *B*. *balangensis* had glabellar furrows, and the effacement of the glabellar furrow is an apomorphy. In fact, the effacement of the glabellar furrow is a plesiomorphy according to the clade *Balangia balangensis*–*Duyunaspis duyunensis*–*Duyunaspis jianheensis* (Fig. 6).

The shape of the glabellar changes gradually from parallel on both sides to middle expansion in the clade *Balangia balangensis*–*Duyunaspis duyunensis*–*Duyunaspis jianheensis* (Fig. 6). Also, the anterior border of *B*. *balangensis*–*D*. *duyunensis*–*D*. *jianheensis* followed a shortening (sag.) trend (Fig. 6). The gradual changes (*B*. *balangensis*–*D*. *duyunensis*–*D*. *jianheensis*) in the shape of the glabellar and the anterior border are both representation of ontogenetic sequences and possibly evidence of phylogeny. In addition, *D*. *duyunensis* and *D*. *jianheensis* both changed progressively from isopygous to micropygous during the ontogeny (Fig. 6). *Balangia*–*Duyunaspis* changes from isopygous to micropygous. The micropygous condition is dominant in the late stratigraphic range of oryctocephalid trilobites (Wuliuan, Miaolingian), while the isopygous condition (*e.g.*, *Arthricocephalus chauveaui*) is more likely to occur in the early stratigraphic range (Stage 4, Series 2) (Yuan et al., 2002). The number of micropygous is more than that of isopygous in the later stratigraphic range of oryctocephalid trilobites, indicating that the micropygous may be advantageous for survival. Both *B*. *balangensis* and *D*. *duyunensis* show a change in the facial suture type during ontogeny, and this change is directional in the evolutionary changes in development. As is expressed (Fig. 6), it changes from *B*. *balangensis* (proparian–gonatoparian) to *D*. *duyunensis* (proparian–gonatoparian–opisthoparian). In fact, this change is the result of the intersection between the end of the posterior branch of the facial suture and the cephalon from the lateral margin of the head, which progressively approaches to the posterior margin of the cephalon. Generally, the facial suture type remained the same in ontogeny. Considering that *D*. *duyunensis* is largely variable in the facial suture type during the ontogeny (Dai et al., 2017), the variation in facial sutures needs to be further confirmed by obtaining more specimens with opisthoparian. In the clade *B*. *balangensis*–*D*. *duyunensis*–*D*. *jianheensis* (Fig. 6), the proparian of *D*. *jianheensis* seemingly remained in the early ontogeny of *B*. *balangensis* or *D*. *duyunensis*. The facial suture type in trilobites is linked to the mode of moulting (Henningsmoen, 1975; Whittington, 1997b; Daley & Drage, 2016). Further studies are needed to determine whether the changes in the moulting patterns of *B*. *balangensis*, *D*. *duyunensis* and *D*. *jianheensis* are related to the changes in facial sutures during its ontogeny and evolution.

**Figure 8:**
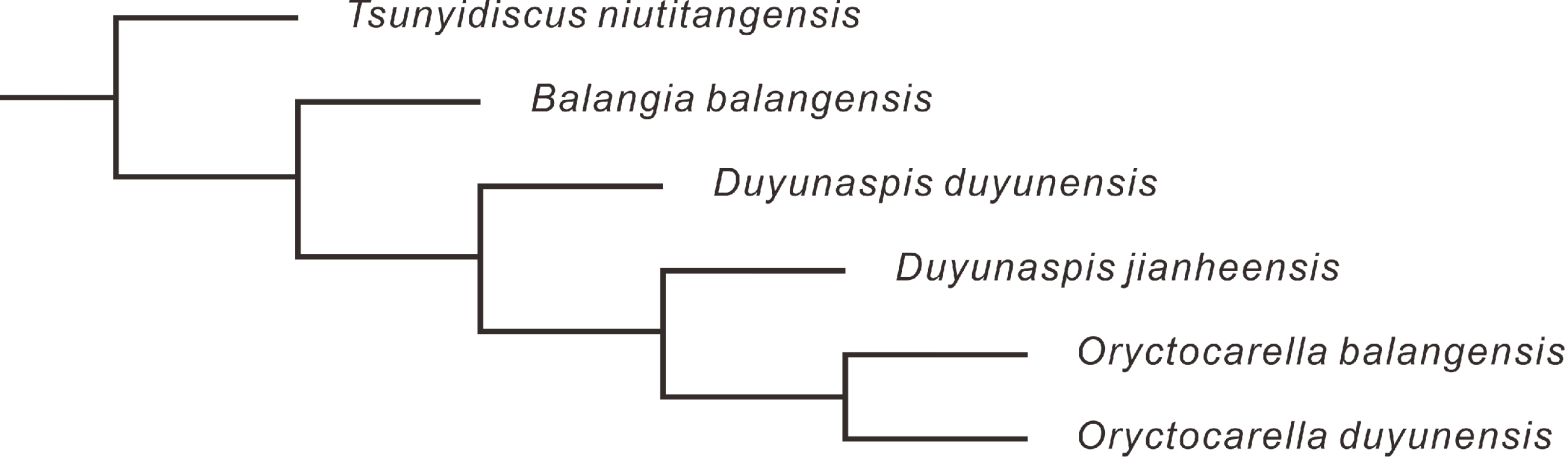
Phylogenetic tree of *Balangia balangensis*, *Duyunaspis duyunensis* and *D*. *jianheensis*. Tree is generated by TNT v1.5 (Goloboff, Farris & Nixon, 2008). A traditional search algorithm (TBR) with 10,000 replications, 1 random seed, and 10 trees saved per replication was used to determine the most parsimonious trees for the data matrix. All characters were unweighted and all multistate characters were treated as unordered. Parsimony analysis recovered one most parsimonious trees of length 42 steps (CI = 0.85, RI = 0.667). For character data see supporting material (see S3 in Supplementary Information).

Yuan, Zhao & Li (2001), Yuan et al. (2002) and McNamara, Yu & Zhou (2006) suggested that *Balangia* might have derived from *Duyunaspis* based on heterochrony. As a potential pattern for an evolutionary change, the key issue in heterochrony is to determine the ancestral-descendant relationships and the timing of sexual maturation (McKinney, 1988; McKinney & McNamara, 1991). McNamara, Yu & Zhou (2006) equated the attainment of sexual maturity with the start of the holaspid period in *B*. *balangensis* and *D*. *duyunensis*, but there was no sufficient evidence to confirm the correlation between sexual maturation of trilobite and its holaspid period (Hughes, Minelli & Fusco, 2006). Based on the changes in growth trajectories, the onset of sexual maturity in the trilobite *Estaingia bilobata* is thought to coincide with the onset of the holaspid period (Holmes, Paterson & García-Bellido, 2021b). However, the changes in the growth trajectory of *E*. *bilobata* do not exactly coincide with the onset of the holaspid period (Holmes, Paterson & García-Bellido, 2021b). In living arthropods, the stage at which sexual maturity begins is not necessarily correlated with the stage at which the number of adult segments is acquired (Minelli & Fusco, 2013). Furthermore, sexual maturation is more than one stage in some modern arthropods, and there are mature stages alternating with intercalary stages, in such stage reproduction not workable in some arthropods (Verhoeff, 1939; Sahli, 1985; Sahli, 1989; Sahli, 1990; Minelli & Fusco, 2013). More evidence is needed to support whether there is a link between sexual maturity and holaspid period of trilobites. Based on the higher strata of *B*. *balangensis* than that of *D*. *duyunensis* in Banglang Formation, Yuan, Zhao & Li (2001), Yuan et al. (2002) and McNamara, Yu & Zhou (2006) suggested that *Balangia* was the descendant of *Duyunaspis*, which is not rigorous because some other factors like incomplete records of fossils or insufficient excavation can influence the first appeared datum. Phylogenetic trees provide a means to determine the ancestral relationships (*e.g.*, Goloboff, 2022). To further verify the relationship between *Balangia* and *Duyunaspis* we selected 25 characters (see S3 in Supplementary Information) and constructed a phylogenetic tree by taking *Tsunyidiscus niutitangensis* as the outgroup (Fig. 8). The phylogenetic tree verified the evolutionary direction of *B*. *balangensis*–*D*. *duyunensis*–*D*. *jianheensis*.

*Balangia balangensis* sporadically distributed in a few strata, accounting for one thousandth of *Duyunaspis duyunensis* in number collected from the Balang Formation. Moreover, the geographical distribution of *B*. *balangensis* is much narrower than that of *D*. *duyunensis* (Qian, 1961; McNamara, Yu & Zhou, 2006; Lei & Peng, 2014; Peng, 2020). Nearly all the early oryctocephalid trilobites we observed were first appeared at the base of Balang Formation in south China, equal to the lowest stratigraphic stratum of oryctocephalid (except Cheiruroidinae, see Peng et al., 2018) trilobites we have identified so far. For this, it is possible that oryctocephalid (except cheiruroidin) trilobites might exist in the strata underlying Balang Formation, and we failed to find it because of the lack of fossil records or excavation. Allmon & Sampson (2016) proposed four common elements in the speciation process: (1) isolated populations must be formed; (2) the new species must survive in isolation; (3) the new population must be genetically differentiated by heredity; (4) a new species must stabilize or expand its population. Both *B*. *balangensis* and *D*. *duyunensis* have been confirmed as two separate species (Qian, 1961; Zhou et al., 1977; Peng, 2020; Chen et al., 2022), and thus both of them meet the requirements in (1) and (3). If *B*. *balangensis* evolved from *D*. *duyunensis* (by heterochrony), the co-occurrence of *B*. *balangensis* and *D*. *duyunensis* in the same strata in Banglang Formation is clearly incompatible with the second element proposed by Allmon & Sampson (2016). And this is also a controversial issue in sympatric speciation (Felsenstein, 1981; Futuyma & Kirkpatrick, 2017). The validity of *B*. *balangensis* and *D*. *duyunensis* (Chen et al., 2022) implies that the time, *D*. *duyunensis* diverged from *B*. *balangensis*, was earlier than their co-existence record. Besides, the earliest fossil records of *D*. *duyunensis* has not been found to co-occur with *B*. *balangensis* in the Balang Formation. Therefore, we speculate that the “original *D*. *duyunensis*” diverged from *B*. *balangensis* and then settled in new habitat, forming the *D*. *duyunensis*. After that, *B*. *balangensis* may have migrated to the habitat of *D*. *duyunensis* from where it lived. The absence of *B*. *balangensis* beneath the stratum where *D*. *duyunensis* occurs may be the result of incompleteness of fossil record. In addition, *B*. *balangensis* of the Balang Formation is extremely rare, which does not accord with the fourth element proposed by Allmon & Sampson (2016). Such rarity may reflect the fact that *B*. *balangensis* here is in the final stages of its stratigraphic range. To sum up, we speculate that *B*. *balangensis* did not evolved (heterochrony) from *D*. *duyunensis*. The developmental evolutionary changes of phenotypic traits appear to be directional in *B*. *balangensis*, *D*. *duyunensis*, and *D*. *jianheensis*, therefore, we believe that the clade *B. balangensis*–*D*. *duyunensis*–*D*. *jianheensis* (Fig. 6) is reasonable.

## Conclusions

There is a directional gradual evolution of *Balangia balangensis*–*Duyunaspis duyunensis*–*Duyunaspis jianheensis* in terms of the cephalon morphology, size of the pygidium relative to the cephalon and the trunk segments number in developmental evolutionary change. Furthermore, according to the four common elements in the speciation process, the lowest datum of *B*. *balangensis* is higher than that of *D*. *duyunensis*, which is probably caused by the incompleteness of the fossil record. We speculate that *Balangia* is not necessarily the descendant of *Duyunaspis*, on the contrary, *Balangia* may be the ancestor of *Duyunaspis*. This inference is also supported by the phylogenetic tree.

##  Supplemental Information

10.7717/peerj.15068/supp-1 Supplemental Information 1 Duyunaspis jianheensis

10.7717/peerj.15068/supp-2 Supplemental Information 2 Balangia balangensis

10.7717/peerj.15068/supp-3 Supplemental Information 3 Character data

10.7717/peerj.15068/supp-4 Supplemental Information 4 The locations and accessions of the 250 specimens

## Additional Information and Declarations

### Data Availability

The following information was supplied regarding data availability: The raw measurements and the locations of the 250 specimens are available in the Supplemental Files.
