# Supplementary material for: Life cycle evolution in the trilobites Balangia and Duyunaspis from the Cambrian Series 2 (Stage 4) of South China
Source: PeerJ. 2023 Apr 10;11:e15068. doi: 10.7717/peerj.15068 (PMC10100804; doi:10.7717/peerj.15068)
Supplement: Supplemental Information 3 [file peerj-11-15068-s003.docx]

**Data matrix used in phylogenetic analyses. Character numbers are shown at the top of the table.**

|  | **1** | **2** | **3** | **4** | **5** | **6** | **7** | **8** | **9** | **10** | **11** | **12** | **13** | **14** | **15** | **16** | **17** | **18** | **19** | **20** | **21** | **22** | **23** | **24** | **25** |
| --- | --- | --- | --- | --- | --- | --- | --- | --- | --- | --- | --- | --- | --- | --- | --- | --- | --- | --- | --- | --- | --- | --- | --- | --- | --- |
| ***Balangia balangensis*** | 1 | 1 | 0 | 0 | 0 | 0 | 0 | 0 | 0 | 1 | 0 | 2 | 1 | 1 | 1 | 1 | 1 | 0 | 0 | 1 | 1 | 0 | 7 | 7 | 1 |
| ***Duyunaspis duyunensis*** | 2 | 1 | 0 | 1 | 0 | 1 | 2 | 1 | 0 | 2 | 2 | 2 | 1 | 2 | 1 | 2 | 1 | 1 | 0 | 1 | 0 | 1 | 6 | 3 | 0 |
| ***Duyunaspis jianheensis*** | 2 | 0 | 1 | 1 | 1 | 1 | 2 | 2 | 0 | 2 | 1 | 2 | 1 | 2 | 0 | 0 | 1 | 1 | 0 | 0 | 0 | 1 | 7 | 4 | 0 |
| ***Tsunyidiscus niutitangensis*** | 0 | 0 | 2 | 1 | 1 | 1 | 1 | 0 | 0 | 0 | 0 | 1 | 2 | 2 | 1 | 0 | 1 | 0 | 1 | 1 | 1 | 0 | ？ | 5 | 1 |
| ***Oryctocarella duyunensis*** | 2 | 0 | 0 | 0 | 1 | 1 | 2 | 1 | 1 | 2 | 2 | 2 | 2 | 0 | 1 | 0 | 0 | 1 | 2 | 0 | 0 | 1 | 7 | 3 | 0 |
| ***Oryctocarella balangensis*** | 2 | 0 | 0 | 0 | ？ | 1 | 2 | 1 | 1 | 2 | 2 | 2 | 2 | 1 | 1 | 0 | 1 | 1 | 1 | 0 | 0 | 1 | ? | 3 | 0 |

**Characters and character states used in phylogenetic analyses**

Cephalon

1. Anterior border. (0) band, (1) thin strip, (2) thread.
2. Proportion of the cephalon that is glabella. (0) < 30%, (1) approximately 30%, (2) > 30%.
3. Glabellar shape. (0) parallel sided, (1) middle expansion, (2) tapered, (3) expands anteriorly.
4. Widest point of glabella. (0) same width, (1) L1, (2) L2 or L3, (3) L4.
5. Glabellar furrows in the early ontogeny. (0) absent, (1) present.
6. Glabellar furrows in holaspid period. (0) absent, (1) present.
7. Character of glabellar furrows. (0) absent, (1) weakly incised, (2) strongly incised.
8. Shape of S1, S2 and S3. (0) absent, (1) pit-like, (2) slit-like.
9. The transverse furrow between the glabellar furrows. (0) absent, (1) present
10. Occipital furrow (S0). (0) absent, (1) weak, (2) strong.
11. Eye ridge in holaspid period. (0) absent, (1) weak, (2) strong.
12. Anterior most position of the eye. (0) posterior of the glabellar, (1) middle of the glabella, (2) anterior of the glabella.
13. Position of center of eye (or eye notch). (0) posterior of the glabellar, (1) middle of the glabella, (2) anterior of the glabella.
14. Palpebral lobe length / glabellar length (including occipital ring). (0) < 30%, (1) 30% – 40%, (2) > 40%.
15. Palpebral area width / glabellar width. (0) < 50%, (1) ≥ 50%.
16. Type of facial suture. (0) proparian, (1) gonatoparian, (2) opisthoparian.
17. Cephalic length (include occipital spine) / cephalic width. (0) < 50%, (1) ≥ 50%.

Thorax

1. Maximum number of thoracic segments. (0) ≤ 4, (1) > 4.
2. Width of pleura / maximum width of thoracic axis (trans.). (0) ≤ 1, (1) between 1 – 1.5, (2) ≥ 1.5.
3. Facet. (0) absent, (1) present.

Pygidium

1. Interpleural furrow. (0) visible, (1) very faint or absent.
2. Inter-ring furrow. (0) absent, (1) present.
3. Maximum number of pygidial segments. (0) 1, (1) 2, (2) 3, (3) 4, (4) 5, (5) 6, (6) 7.
4. Maximum number of pygidial segments in holaspid period. (0) 1, (1) 2, (2) 3, (3) 4, (4) 5, (5) 6, (6) 7.
5. Pygidium size relative to cephalon in holaspid period. (0) micropygous, (1) isopygous.
