# Supplementary material for: Life cycle evolution in the trilobites Balangia and Duyunaspis from the Cambrian Series 2 (Stage 4) of South China
Source: PeerJ. 2023 Apr 10;11:e15068. doi: 10.7717/peerj.15068 (PMC10100804; doi:10.7717/peerj.15068)
Supplement: Supplemental Information 4 [file peerj-11-15068-s004.docx]

*Duyunaspis* *jianheensis* (S1 in Supplementary Information). All the specimens are housed at the Guizhou Research Center for Palaeontology (GRCP), Guiyang City, Guizhou Province, China.

|  | **accession numbers** | **storage location** |
| --- | --- | --- |
| 1 | Q51-2434-2 | GRCP |
| 2 | Q52-2060B-4 | GRCP |
| 3 | Q51-2995-3 | GRCP |
| 4 | Q51-2995-4 | GRCP |
| 5 | Q52-2060B-5 | GRCP |
| 6 | Q52-1462 | GRCP |
| 7 | Q52-1594 | GRCP |
| 8 | Q52-2055-2 | GRCP |
| 9 | Q52-2060A-4 | GRCP |
| 10 | Q52-2060A-8 | GRCP |
| 11 | Q51-1836 | GRCP |
| 12 | Q52-1504-2 | GRCP |
| 13 | Q52-3130 | GRCP |
| 14 | Q51-1882 | GRCP |
| 15 | Q51-3257 | GRCP |
| 16 | Q52-239 | GRCP |
| 17 | Q52-1761 | GRCP |
| 18 | Q52-1845-3 | GRCP |
| 19 | Q52-1950 | GRCP |
| 20 | Q52-2060B-7 | GRCP |
| 21 | Q52-2728-2 | GRCP |
| 22 | Q52-3341 | GRCP |
| 23 | Q51-87 | GRCP |
| 24 | Q51-2301 | GRCP |
| 25 | Q51-2813 | GRCP |
| 26 | Q52-2147-2 | GRCP |
| 27 | Q52-3621 | GRCP |
| 28 | Q51-846 | GRCP |
| 29 | Q51-1028 | GRCP |
| 30 | Q51-1120 | GRCP |
| 31 | Q51-1328-5 | GRCP |
| 32 | Q51-1403 | GRCP |
| 33 | Q51-1920 | GRCP |
| 34 | Q51-2393 | GRCP |
| 35 | Q51-2418 | GRCP |
| 36 | Q51-2434-1 | GRCP |
| 37 | Q51-2926 | GRCP |
| 38 | Q51-3077 | GRCP |
|  | **accession numbers** | **storage location** |
| 39 | Q51-3186 | GRCP |
| 40 | Q52-A0 | GRCP |
| 41 | Q52-172 | GRCP |
| 42 | Q52-304-2 | GRCP |
| 43 | Q52-523 | GRCP |
| 44 | Q52-846 | GRCP |
| 45 | Q52-1845-2 | GRCP |
| 46 | Q52-1952-2 | GRCP |
| 47 | Q52-2060A-3 | GRCP |
| 48 | Q52-2084 | GRCP |
| 49 | Q52-75-2 | GRCP |
| 50 | Q51-1166 | GRCP |
| 51 | Q51-2255 | GRCP |
| 52 | Q51-2356 | GRCP |
| 53 | Q51-2993 | GRCP |
| 54 | Q51-5001 | GRCP |
| 55 | Q52-262 | GRCP |
| 56 | Q52-888 | GRCP |
| 57 | Q52-1482-2 | GRCP |
| 58 | Q52-1510 | GRCP |
| 59 | Q52-1586 | GRCP |
| 60 | Q52-2060B-8 | GRCP |
| 61 | Q52-2064 | GRCP |
| 62 | Q52-2301 | GRCP |
| 63 | Q52-2775-3 | GRCP |
| 64 | Q51-1835 | GRCP |
| 65 | Q51-2752 | GRCP |
| 66 | Q51-3233 | GRCP |
| 67 | Q52-921 | GRCP |
| 68 | Q52-A1 | GRCP |
| 69 | Q51-1337 | GRCP |
| 70 | Q51-1498 | GRCP |
| 71 | Q51-2160 | GRCP |
| 72 | Q51-2229 | GRCP |
| 73 | Q51-2418-2 | GRCP |
| 74 | Q51-2441 | GRCP |
| 75 | Q51-2756 | GRCP |
| 76 | Q51-2849 | GRCP |
|  | **accession numbers** | **storage location** |
| 77 | Q51-3026 | GRCP |
| 78 | Q51-3168 | GRCP |
| 79 | Q51-3246 | GRCP |
| 80 | Q52-95 | GRCP |
| 81 | Q52-518 | GRCP |
| 82 | Q52-1140 | GRCP |
| 83 | Q52-1164 | GRCP |
| 84 | Q52-1251 | GRCP |
| 85 | Q52-1504 | GRCP |
| 86 | Q52-1528 | GRCP |
| 87 | Q52-1563 | GRCP |
| 88 | Q52-1594-2 | GRCP |
| 89 | Q52-1619 | GRCP |
| 90 | Q52-1629 | GRCP |
| 91 | Q52-1671 | GRCP |
| 92 | Q52-1728 | GRCP |
| 93 | Q52-1730 | GRCP |
| 94 | Q52-1785 | GRCP |
| 95 | Q52-1952-1 | GRCP |
| 96 | Q52-2060B-1 | GRCP |
| 97 | Q52-2261 | GRCP |
| 98 | Q52-3147 | GRCP |
| 99 | Q52-3204 | GRCP |
| 100 | Q52-3544 | GRCP |
| 101 | Q51-396 | GRCP |
| 102 | Q51-932 | GRCP |
| 103 | Q51-1111 | GRCP |
| 104 | Q51-1347 | GRCP |
| 105 | Q51-1550 | GRCP |
| 106 | Q51-2578 | GRCP |
| 107 | Q51-2946 | GRCP |
| 108 | Q51-3263 | GRCP |
| 109 | Q51-A2 | GRCP |
| 110 | Q52-66 | GRCP |
| 111 | Q52-66-2 | GRCP |
| 112 | Q52-304-1 | GRCP |
| 113 | Q52-304-3 | GRCP |
| 114 | Q52-469 | GRCP |
| 115 | Q52-719 | GRCP |
| 116 | Q52-749 | GRCP |
| 117 | Q52-1145 | GRCP |
|  | **accession numbers** | **storage location** |
| 118 | Q52-1210-2 | GRCP |
| 119 | Q52-1486 | GRCP |
| 120 | Q52-1957 | GRCP |
| 121 | Q52-2055 | GRCP |
| 122 | Q52-2147 | GRCP |
| 123 | Q52-2613 | GRCP |
| 124 | Q52-3196 | GRCP |
| 125 | Q52-3665 | GRCP |
| 126 | Q52-45 | GRCP |
| 127 | Q52-74 | GRCP |
| 128 | Q52-85-1 | GRCP |
| 129 | Q51-2995-1 | GRCP |
| 130 | Q52-2060A-1 | GRCP |
| 131 | Q52-2836-1 | GRCP |
| 132 | Q52-3727 | GRCP |
| 133 | Q51-1593 | GRCP |
| 134 | Q52-A3 | GRCP |
| 135 | Q52~A4 | GRCP |
| 136 | Q52-809 | GRCP |
| 137 | Q52-1418 | GRCP |
| 138 | Q52-2137 | GRCP |
| 139 | Q52-3920 | GRCP |
| 140 | Q51-A5 | GRCP |
| 141 | Q51-230 | GRCP |
| 142 | Q51-248 | GRCP |
| 143 | Q51-920 | GRCP |
| 144 | Q51-1122 | GRCP |
| 145 | Q51-1744 | GRCP |
| 146 | Q51-1818 | GRCP |
| 147 | Q51-1976 | GRCP |
| 148 | Q51-2117 | GRCP |
| 149 | Q51-2500 | GRCP |
| 150 | Q51-3041 | GRCP |
| 151 | Q52-43-1 | GRCP |
| 152 | Q52-140 | GRCP |
| 153 | Q52-229 | GRCP |
| 154 | Q52-348 | GRCP |
| 155 | Q52-445 | GRCP |
| 156 | Q52-968 | GRCP |
| 157 | Q52-1345 | GRCP |
| 158 | Q52-1347 | GRCP |
|  | **accession numbers** | **storage location** |
| 159 | Q52-1482 | GRCP |
| 160 | Q52-1598 | GRCP |
| 161 | Q52-2060B-2 | GRCP |
| 162 | Q52-2232 | GRCP |
| 163 | Q52-2554 | GRCP |
| 164 | Q52-2572 | GRCP |
| 165 | Q52-2775-1 | GRCP |
| 166 | Q52-2775-2 | GRCP |
| 167 | Q52-3836-2 | GRCP |
| 168 | Q52-3721 | GRCP |
| 169 | Q52-4185 | GRCP |
| 170 | Q52-62 | GRCP |
| 171 | Q52-75 | GRCP |
| 172 | Q51-72 | GRCP |
| 173 | Q51-243 | GRCP |
| 174 | Q51-807B-2 | GRCP |
| 175 | Q51-1328-1 | GRCP |
| 176 | Q51-1328-2 | GRCP |
| 177 | Q51-1528 | GRCP |
| 178 | Q51-1931 | GRCP |
| 179 | Q51-2180 | GRCP |
| 180 | Q51-2441-2 | GRCP |
| 181 | Q51-2451-4 | GRCP |
| 182 | Q51-2995-2 | GRCP |
| 183 | Q51-3109-1 | GRCP |
| 184 | Q51-3267 | GRCP |
| 185 | Q51-3543 | GRCP |
| 186 | Q52-A6 | GRCP |
| 187 | Q52-164 | GRCP |
| 188 | Q52-715 | GRCP |
| 189 | Q52-1185 | GRCP |
| 190 | Q52-1250 | GRCP |
| 191 | Q52-1931-2 | GRCP |
| 192 | Q52-2060B-6 | GRCP |
| 193 | Q52-2233 | GRCP |
| 194 | Q52-2308 | GRCP |
| 195 | Q52-2496 | GRCP |
| 196 | Q52-2583 | GRCP |
| 197 | Q52-2612 | GRCP |
| 198 | Q52-2688 | GRCP |
| 199 | Q52-2728 | GRCP |
|  | **accession numbers** | **storage location** |
| 200 | Q52-2991 | GRCP |
| 201 | Q52-3309 | GRCP |
| 202 | Q52-3694 | GRCP |
| 203 | Q51-1021B | GRCP |
| 204 | Q51-1021B-2 | GRCP |
| 205 | Q51-2302 | GRCP |
| 206 | Q51-3004 | GRCP |
| 207 | Q52-1554 | GRCP |
| 208 | Q52-3271 | GRCP |
| 209 | Q52-3318B | GRCP |
| 210 | Q52-4173 | GRCP |
| 211 | Q52-85-2 | GRCP |
| 212 | Q51-1605 | GRCP |
| 213 | Q51-3202 | GRCP |
| 214 | Q52-1198 | GRCP |
| 215 | Q52-1357 | GRCP |
| 216 | Q52-1845-1 | GRCP |
| 217 | Q52-1684 | GRCP |
| 218 | Q52-2474 | GRCP |
| 219 | Q52-2499 | GRCP |
| 220 | Q221-A7 | GRCP |
| 221 | Q51-268 | GRCP |
| 222 | Q51-499B | GRCP |
| 223 | Q51-0101 | GRCP |
| 224 | Q51-807B-1 | GRCP |
| 225 | Q51-2504 | GRCP |
| 226 | Q52-A8 | GRCP |
| 227 | Q52-3467A | GRCP |
| 228 | Q51-1142 | GRCP |
| 229 | Q51-2451 | GRCP |
| 230 | Q51-2823 | GRCP |
| 231 | Q52-1827 | GRCP |
| 232 | Q52-3659 | GRCP |
| 233 | Q51-1834-2 | GRCP |
| 234 | Q52-1525 | GRCP |
| 235 | Q52-2224A-4 | GRCP |
| 236 | Q52-3868 | GRCP |

*Balangia* *balangensis* (S2 in Supplementary Information). All the specimens are housed at the Guizhou Research Center for Palaeontology (GRCP), Guiyang City, Guizhou Province, China.

|  | **accession numbers** | **storage location** |
| --- | --- | --- |
| 1 | JJB-B-19-1 | GRCP |
| 2 | JJB-B-18 | GRCP |
| 3 | JJB-B-5 | GRCP |
| 4 | JJB-B-6 | GRCP |
| 5 | JJB-B-9 | GRCP |
| 6 | JJB-B-13 | GRCP |
| 7 | JJB-B-21 | GRCP |
| 8 | JJB-B-123 | GRCP |
| 9 | JJB-B-3 | GRCP |
| 10 | JJB-B-4 | GRCP |
| 11 | JJB-B-12-3 | GRCP |
| 12 | JJB-B-15 | GRCP |
| 13 | JJB-B-22 | GRCP |
| 14 | JJB-B-12-1 | GRCP |
